# Phytochemical Screening and In Vitro Antifungal Activity of Selected Medicinal Plants against *Candida albicans* and *Aspergillus niger* in West Shewa Zone, Ethiopia

**DOI:** 10.1155/2022/3299146

**Published:** 2022-06-28

**Authors:** Askale Gizaw, Lencho Megersa Marami, Ibsa Teshome, Edilu Jorga Sarba, Petros Admasu, Dagmawit Atalel Babele, Getachew Mulatu Dilba, Wakuma Mitiku Bune, Morka Dandecha Bayu, Miressa Tadesse, Kebede Abdisa

**Affiliations:** ^1^Department of Veterinary Laboratory Technology, School of Veterinary Medicine, Guder Mamo Mezemir Campus, Ambo University, P.O. Box 19, Ambo, Oromia, Ethiopia; ^2^Department of Veterinary Science, School of Veterinary Medicine, Guder Mamo Mezemir Campus, Ambo University, P.O. Box 19, Ambo, Oromia, Ethiopia; ^3^Department of Chemistry, College of Computational and Natural Science, Ambo University, P.O. Box 19, Ambo, Oromia, Ethiopia

## Abstract

Antimicrobial resistance to commercially available medications has become a global issue, yet there is still the possibility of developing new drugs from medicinal plants. As a result, the aims of the present study were to screen secondary metabolites and to evaluate in vitro antifungal activities of *Brucea antidysenterica*, *Aloe vera,* and *Justicia schimperiana*. After the plants were identified, their leaves were collected, washed, dried under the shade, pulverized, and extracted with methanol (99.8%) using the maceration technique. The presence of secondary metabolites in plant extracts was screened using various laboratory protocols. The antifungal activities of the plant extract against reference fungal strains of *Candida albicans* and *Aspergillus niger* at concentrations of 200, 100, and 50 mg/mL were assessed using the agar-well diffusion method. Ketoconazole (15 *μ*g) was used as a positive control, while 5% dimethyl sulfoxide and/or 5% Tween 80 were used as negative controls. All tests were conducted in triplicate. Alkaloids, flavonoids, and phenols were secondary metabolites found in all plant extracts. The extract of leaves of *B. antidysenterica* and *J. schimperiana* formed a mean zone of inhibition of 15.5 ± 0.5 mm and 15.3 ± 0.58 mm, respectively, against *Candida albicans* at a concentration of 200 mg/mL, whereas extracts of *A. vera* leaves formed a 12.3 ± 0.58 mm inhibition zone only against *Aspergillus niger* at 200 mg/mL. In conclusion, the current study found that *B. antidysenterica*, *A. vera,* and *J. schimperiana* had antifungal activity. In addition, all these plants had a variety of secondary metabolites that possibly have antifungal activities. Studies on in vivo investigations and isolation of specific antifungal compounds from these medicinal plants are suggested.

## 1. Introduction

Medicinal plants are used to treat different microbial infections. The World Health Organization reports that various plant fractions and their dynamic constituents are used as traditional medicines by 80% of the world population [1–5]. In Ethiopia, 90% of the medicine of the livestock and 70% of human population depend on traditional medicine [6]. Therapeutic usefulness of plants is decided by their chemical contents or phytochemical ingredients, which are present naturally in plants [7, 8]. Flavonoids, alkaloids, tannins, saponins, phenols, and glycosides are the major secondary metabolites found in plants that have antioxidant, antiinflammatory, anticancer, and antimicrobial properties [9].

*Brucea antidysenterica* (Qomonyo in Afan Oromo) is a shrub or small tree that is 10 to 15 m high. *B. antidysenterica* is a well-known *Brucea* species that is widely grown in Ethiopia [10]. Various parts of *B. antidysenterica* are used in traditional medicine for different purposes. The leaves of *B. antidysenterica* have a wound-healing effect [11]. The roots of this plant also have antibacterial activities [12].

*Justicia schimperiana* (Dhumuuga in Afan Oromo) is a shrub with branched stems that belongs to the family *Acanthaceae*. In various parts of Ethiopia, *J. schimperiana* is used to treat animal and human ailments. Traditionally, *J. schimperiana* has been used for the treatment of diabetes mellitus [13], liver disease [14], rabies in humans, blackleg, internal parasites in livestock [15], malaria, gonorrhoea [16], and coccidiosis [17].

*Aloe vera* (Hargisa in Afan Oromo) is a plant belonging to the genus *Aloe*. The leaves of *A. vera* are triangular with serrated edges. *A. vera* is used as a traditional remedy for a variety of conditions. *A. vera* is a useful plant in treating various diseases such as arthritis, eye disease, type II diabetes, tumors, liver complaints, spleen enlargement, bronchitis, vomiting, asthma, jaundice ulcers, and wounds [18,19].

*Candida albicans* is the most virulent *Candida* species in the genus *Candida* that causes diseases called candidiasis in humans and animals [20]. Domestic animals such as cattle, horses, pigs, cats, and dogs as well as birds are susceptible to *Candida* infections [21]. *C. albicans* resides as a harmless commensal in the nasopharynx, GI tract, and external genitalia of many species of animals [20]. Administration of antibiotics and immunosuppressive drugs are some of the factors associated with *Candida* infection [22].

*Aspergillus niger* is a filamentous fungus that grows on organic matter. In nature, it is found in litter, soil, compost, and on decaying plant material [23]. It causes a disease known as aspergillosis or black mold. In animals, aspergillosis is primarily a respiratory infection that may become generalized. However, tissue predilection is highly variable among species [24]. Some strains of *A. niger* have been reported to produce potent mycotoxins [25].

Antimicrobial resistance to commercially available medications has become a worldwide problem in recent years. Similarly, several species are developing resistance to currently available antifungal medications. This shows that there is a need to investigate other options. In terms of confirming traditional usage and assessing phytochemical content, medicinal plants have become the focus of significant research. Because of its accessibility and affordability, Ethiopia has a long history of using a traditional health care system. Considering the need for alternative fungicides, it was believed to be useful to appraise the antifungal effects of locally available plant extracts. Antifungal activity and phytochemical constituents of a few medicinal plants were investigated and reported in Ethiopia [26–28].

Various ethnobotanical surveys of medicinal plants used to treat fungal infections have been reported in Ethiopia by various researchers [29–33]. A botanical survey conducted in Horo Guduru, Western Ethiopia, showed that *B. antidysenterica* has been used by traditional healers to treat fungal infections [17]. Traditional usage of *Aloe* species to treat fungal infections was reported in Hadiya Zone, Ethiopia [29]. The antifungal activities of *A. vera* in Ethiopia have not been confirmed experimentally. Although research on the antifungal activities of *B. antidysenterica* and *J. schimperiana* has been done in other parts of Ethiopia, it has not been done in the current study area. Therefore, the aims of the present study were to screen secondary metabolites and evaluate in vitro antifungal activities of methanol extracts of *B. antidysenterica*, *A. vera*, and *J. schimperiana* against *C. albicans* and *A. niger.*

## 2. Materials and Methods

### 2.1. Description of Plant Collection Areas

**Figure 1:**
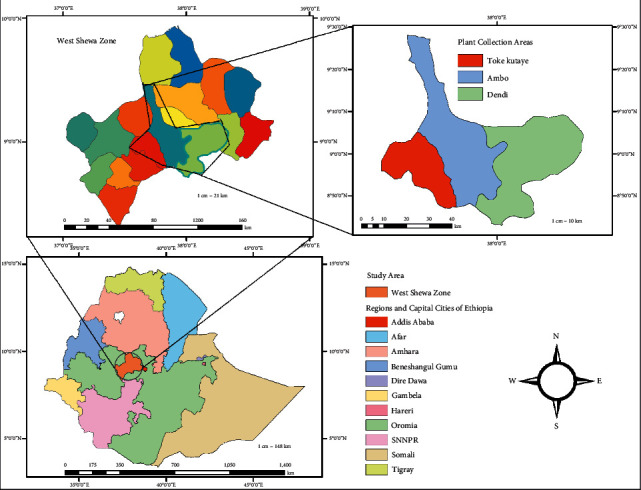
Map of the study area.

Leaves of *B. antidysenterica*, *A. vera*, and *J. schimperiana* were collected from August 2018 to October 2018, respectively, from Ambo, Toke Kutaye, and Dendi districts in West Shewa Zone Oromia Regional State, Ethiopia. The Dendi, Ambo, and Toke Kutaye districts are found 79 km, 114 km, and 162 km west of Addis Ababa, respectively (Figure 1). The traditional knowledge and practice of ethnomedicine in these areas are well known. Dendi, Ambo, and Toke Kutaye are in the center of the country and have elevations ranging from 2000 to 3288 m, 1900 to 2275 m, and 1580 to 3194 m, respectively. The annual temperature of the Dendi district ranges from 9.3°C to 23.8°C, while it is 10°C to 29°C in the Ambo and Toke Kutaye districts [34,35].

### 2.2. Study Design

A laboratory experiment was conducted to evaluate the antifungal activities of *B. antidysenterica, A. vera,* and *J. schimperiana* against *C. albicans* and *A. niger* using the agar-well diffusion method.

### 2.3. Plant Collection, Authentication, and Preparation

Fresh leaves of *B. antidysenterica*, *A. vera,* and *J. schimperiana* were collected from three districts of the West Shewa Zone after being named and authenticated by a botanist, Biruk Bedore, Department of Forestry, Ambo University. The voucher numbers given for *B. antidysenterica, A. vera,* and *J. schimperiana* were AUH/005/2018, AUH/006/2018, and AUH/008/2018, respectively. The collected plants were taken to the Veterinary Microbiology Laboratory at Ambo University, Guder Mamo Mezemir Campus. Leaves of these plants were then thoroughly cleansed with distilled water to remove dust and dirt particles. The leaves of *A. vera, J. schimperiana,* and *B. antidysenterica* were chopped into pieces. Then, all plants were spread on the paper sheet and dried for two weeks in the shade at room temperature and powdered with an electric grinder [36]. Finally, the powder was stored until it was needed for extraction.

### 2.4. Plant Extraction

A maceration technique was used to extract leaves of plants using 99.8% methanol (Sisco Research Laboratories Pvt. Ltd., India) at the Chemistry Department, Ambo University. The powder of plants was soaked in methanol (1 : 4 ratio) in a separate flask and shaken for 72 hrs using an automatic orbital shaker. The suspensions were filtered with Whatman No.1. The filtrate was then concentrated under reduced pressure with a rotary evaporator before being dried in a 40°C oven [36].

The yield of the methanol extracts of all plants was determined. The percentage yield was calculated by dividing the weight of crude obtained after extraction by the weight of plant powder weighed before extraction and multiplied it by 100.

### 2.5. Preliminary Phytochemical Screening

Using various standard laboratory techniques, the presence or absence of secondary metabolites such as phenols, saponins, tannins, alkaloids, flavonoids, and glycosides in each plant extract was checked.

#### 2.5.1. Test for Phenols

Five drops of a 5% neutral ferric chloride solution were added to 0.25 g of each crude extract solution. The formation of a deep blue-black color showed the presence of phenols [37].

#### 2.5.2. Test for Tannins

0.5 g of crude of each plant extract was mixed with 10 mL of distilled water and boiled and then filtered. Three drops of 0.10% ferric chloride were added to the filtrate. The formation of brownish, greenish, or blue-black color was an indication of the presence of tannins [38].

#### 2.5.3. Test for Alkaloids

0.5 g of extract was weighed and added to 10 mL of acid alcohol. After mixing, it was boiled and filtered. A 2 mL dilute ammonia was added to the 5 mL filtrate. To extract the alkaloidal base, 5 mL of chloroform was added. The chloroform layer was extracted with 10 mL of acetic acid. This was split into two portions. Mayer's reagent was added to one portion and Dragendorff's reagent to the other. The formation of a cream by Mayer's reagent or reddish-brown precipitate by Dragendorff's reagent was considered as positive for the presence of alkaloids in each plant extract [38].

#### 2.5.4. Test for Saponins

A 0.25 g crude extract was dissolved in 5 mL of distilled water, shaken, and seen for a stable, persistent froth. The formation of froth was an indication of the presence of saponins [38].

#### 2.5.5. Test for Flavonoids

About 0.25 g of crude extract and 10 mL of ethyl acetate were added to a test tube and heated in a water bath for 3 minutes. The mixture was cooled, filtered, and then approximately 4 mL of the filtrate was taken and shaken with 1 mL of dilute ammonia solution. A yellow coloration indicated the presence of flavonoids [37].

#### 2.5.6. Test for Glycosides

50 mg of each plant extract was hydrolyzed for 2 hrs in a water bath with concentrated hydrochloric acid and filtered, and the hydrolyzed extract was treated according to the legal test. The extract was dissolved in pyridine, sodium nitroprusside solution was added, and the solution was made alkaline with 10% NaOH. The pink color indicated the presence of glycosides [39].

#### 2.5.7. Reference Organisms

*C. albicans* (ATCC 10231) and *A. niger* (ATCC 6275) were standard fungal strains collected from the Microbiology Laboratory of the Ethiopian Public Health Institute (EPHI) in Addis Ababa, Ethiopia, and transported to the laboratory of the Veterinary Laboratory Technology, Ambo University, under the cold chain.

### 2.6. Antibiotic Disks and Dissolvents

A standard disk of the antifungal drug, ketoconazole (15 *µ*g), was used as a positive control. Extracts of *B. antidysenterica* and *J. schimperiana* were dissolved in 5% dimethyl sulfoxide (DMSO) (negative control). Since 5% DMSO cannot dissolve *A. vera* extract, 5% Tween 80 (negative control) was used to dissolve *A. vera*.

### 2.7. Agar-Well Diffusion Method

The agar-well diffusion method was used to evaluate the antifungal activities of the plants [40]. First, *A. niger* colonies that were stored on SDA agar slant were subcultured on SDA plate and incubated at 35°C for three days. *A. niger* colonies that were taken from this fresh culture (3 days old) were mixed with 1 mL of sterile physiological saline solution, and a drop of Tween 20 was used to facilitate and produce an *A. niger* inoculum. After complete dissolution, the inoculum supernatant was compared with the 0.5 McFarland standard and adjusted by physiological solution. The supernatant was used for antifungal tests. *C. albicans* inoculum suspensions were prepared by taking a few colonies from fresh cultures grown on the SDA plate. The colonies were suspended in 5 mL of sterile physiological saline. The inoculum suspensions were shaken till complete dissolution, and the turbidity of the inoculum was compared with the 0.5 McFarland standard and adjusted with sterile physiological saline solution.

The antifungal activities of methanol extract of *B. antidysenterica*, *A. vera,* and *J. schimperiana* were tested using SDA plates. Test organisms were inoculated uniformly with sterile swabs on the surface of the solidified SDA plate. After inoculation, four 6 mm diameter holes were made by using a sterile cork borer. The holes were filled with 0.1 mL of 200, 100, and 50 mg/mL concentrations of the crude extracts, negative control (5% DMSO and 5% Tween 80) using a micropipette, and positive control (Ketoconazole 15 *µ*g). The plates were then left at room temperature for 1 hr for diffusion and incubated. The zone of inhibition produced by *C. albicans* was measured in four directions and recorded after 48 hrs of incubation at 30°C [41]. Instead, the zone of inhibition against *A. niger* was measured after 7 days growth at room temperature. Each test was done in triplicate. Values were given as the mean ± standard deviation (SD) of tests performed in triplicate.

### 2.8. Data Analysis

The data collected were stored in Microsoft Excel and analyzed using statistical software (STATA version 14). A one-way ANOVA was performed to test variation among the groups' mean concentrations of crude extracts. A Tukey post hoc test was used to compare the association between the zone of inhibition among concentration groups and the negative control. *p* value <0.05 was considered as statistically significant difference.

## 3. Results

### 3.1. Percentage Yield of Plant Extracts

The percentage yield of each crude extract was determined and is shown in Table 1. Comparatively, *J. schimperiana* produced a higher yield while *A. vera* had a lower yield.

### 3.2. Preliminary Phytochemical Screening

According to preliminary phytochemical assays, the plants had different secondary metabolites. Except for tannins in *B. antidysenterica*, glycosides in *J. schimperiana*, saponins, and glycosides in *A. vera*, each plant extract had all the secondary metabolites tested. Generally, all the plant extracts had alkaloids, flavonoids, and phenols (Table 2).

### 3.3. Antifungal Activities of Plant Extracts

The in vitro antifungal activities of methanol extracts of *B. antidysenterica*, *A. vera*, and *J. schimperiana* at concentrations of 200 mg/mL, 100 mg/mL, and 50 mg/mL were tested against *C.albicans* and *A. niger* in the present study. The details of the results are shown in Table 3. At a higher concentration (200 mg/mL), the extracts of *B. antidysenterica* and *J. schimperiana* showed higher antifungal activities against *C. albicans* with inhibition zones of 15.5 ± 0.5 mm and 15.3 ± 0.58 mm, respectively, while only *A. vera* plant extract showed antifungal activity against *A. niger* with an inhibition zone of 12.3 ± 0.58 mm.

There was a statistically significant difference between the categories of concentration and the negative control, as determined by one-way ANOVA. A Tukey post hoc test revealed that the zone of inhibition was significantly higher at a higher concentration (200 mg/mL) compared to a lower concentration (50 mg/mL) and negative control (Table 3).

## 4. Discussion

According to phytochemical screening tests, alkaloids, phenols, and flavonoids were secondary metabolites found in the extracts of leaves of *B. antidysenterica, A. vera,* and *J. schimperiana*. In this study, alkaloids, flavonoids, saponins, glycosides, and phenols were found in the methanolic extract of B. antidysenterica leaves. This is consistent with the previous work by Dilnesa et al. [42], who found alkaloids, steroids, saponins, phenols, flavonoids, and glycosides in B. antidysenterica extracted by 80% methanol but not tannins.

Alkaloids, flavonoids, tannins, and phenols were the secondary metabolites found in the methanolic extract of *A. vera* leaves, while saponins and glycosides were not found in the extract of *A. vera*. The present finding is in line with the previous findings of Nalin Pagi et al. [43], who found alkaloids, phenols, and flavonoids in *A. vera* leaf extract but not glycosides. Nalin Pagi et al. [43] also found saponins in the extract which were not found in *A. vera* leaf extract in the present finding. The discrepancy could be attributed to differences in geographical areas and soil content where the plants grow, plant collection seasons, and plant growth stages [44–46].

Alkaloids, flavonoids, saponins, tannins, and phenols were detected in methanolic extract of *J. schimperiana* leaves. The present finding is in line with the previous findings of Mekonnen et al. [47] and Tesera et al. [48], who reported the presence of phenols, tannins, flavonoids, and saponins in *J.schimperiana* leaves extracted with 80% methanol. The variations could be attributed to the differences in solvent concentrations. According to Pandey and Tripathi [49], the concentration of the solvent used affects the solubility of certain plant components. The earlier study used hydromethanolic (80% methanol), which extracts a wider variety of phytochemicals than absolute methanol, which was used in this investigation [36,50].

The antifungal activity of a methanolic extract of *B. antidysenterica* and *J. schimperiana* against *C. albicans* and *A. vera* against *A. niger* was showed in this investigation. Secondary metabolites such as alkaloids, phenols, and flavonoids, which have been reported to have antimicrobial activity, are present in each plant extract. Alkaloids are chemical compounds with a wide range of structures that are reported to have antimicrobial properties by blocking enzyme activity [51]. Flavonoids are structurally diverse secondary metabolites in plants that are reported to inhibit fungal growth by disrupting plasma membranes, inducing mitochondrial malfunction, and reducing cell wall construction, cell division, RNA (Ribonucleic acid), and protein synthesis, as well as the efflux mediated pumping system [52]. Phenols are a group of secondary metabolites distributed in plants that are used as antimicrobial agents due to their potential to damage membrane structural integrity in a nonspecific way and to inhibit certain electron transport enzymes [53].

In the present study, the antifungal activity of a methanolic extract of *B. antidysenterica* leaf extract against *C. albicans* was seen. In the present finding, B. antidysenterica leaf extract had no activity against *A. niger*, which contradicts with the finding of Guluma et al. [54], who reported antifungal activity of *B. antidysenterica* leaf extract against *A. niger*. The variation could be attributed to differences in geographical areas and soil content where the plants grow, plant collection seasons, plant growth stages [44], and extraction methods used [49].

In the present study, *A. vera* leaf extract was found to have antifungal activity against *A. niger* but not against *C. albicans*. Like the present finding, antifungal activities of *A. vera* extract against *A. niger* were reported by Sitara et al. [55].

The antifungal activity of a methanolic extract of *J. schimperiana* leaf was also observed. Tesfaye [56] also reported the antifungal activity of J. schimperiana leaf extract extracted with 80% methanol and found that the extract (14.7 ± 0.3 mm) at 200 mg/mL showed comparable activity to the present finding against *C. albicans*. This finding agreed with a previous report on the anticandidal activity of *J. schimperiana*, which inhibited the growth of *C. albicans* [57]. The phytochemicals found in the methanol extract of *J. schimperiana* could be the reason for its antifungal activity. Among the phytochemicals found in the crude extract of *J. schimperiana,* saponins, tannins, flavonoids, alkaloids, and phenols had been reported to have antifungal activity [51–53]. Tannins are other chemicals discovered in *J. schimperiana* extracts that have been attributed to antimicrobial activity in several studies. Tannins' antimicrobial mechanism could be due to their membrane-damaging effects and metabolic pathway blockage, which could lead to the microorganism's death [58]. Saponins could also contribute to antifungal activity of current plant extract.

The current study's main limitation is that different solvents that have different polarities were not considered, some secondary metabolites were not screened, and minimum inhibitory concentration techniques were not used.

## 5. Conclusions

The current study revealed that there were higher yields of *J. schimperiana* extracts obtained than those of *A. vera* using methanol. The phytochemical screening results showed that alkaloids, flavonoids, and phenols were the secondary metabolites found in all plant extracts, while glycosides were another phytochemical constituents found only in *B. antidysenterica.* All plants showed varying degrees of antifungal activity against fungal strains, which implies that they could be a source of new drugs to treat fungal diseases. Toxicity study and fractionation of plant compounds are the future study plan.

## Figures and Tables

**Table 1:** Results of percentage yield of plant crude extracts with methanol.

Plant species	Weight of crude (*g*)	Weight of sample (*g*)	Yield (%)
*Brucea antidysenterica*	17	215	7.9
*Aloe vera*	4.7	100	4.7
*Justicia schimperiana*	13	151	8.6

**Table 2:** Results of preliminary phytochemical screening tests of methanolic plant extracts.

Name of plants	Secondary metabolites					
	Alkaloids	Phenols	Tannins	Saponins	Flavonoids	Glycosides
*B. antidysenterica*	++	+	−	++	+	+
*A. vera*	+	+	+	−	+	−
*J. schimperiana*	+	+	+	+	+	−

Notes: − absent, + slightly present, and ++ present.

**Table 3:** Antifungal activity of methanolic extracts of leaves of plants against *C. albicans* and *A. niger.*

Categories	Concentration (mg/mL)	*C. albicans*	*A. niger*
*B. antidysenterica*	200	15.5 ± 0.5^ab^	6.0 ± 0.0
*B. antidysenterica*	100	13 ± 1.00^a^	6.0 ± 0.0
*B. antidysenterica*	50	11.3 ± 0.58^a^	6.0 ± 0.0
*A. vera*	200	6.0 ± 0.0	12.3 ± 0.58^ac^
*A. vera*	100	6.0 ± 0.0	11.7 ± 0.58^a^
*A. vera*	50	6.0 ± 0.0	10.3 ± 0.58^a^
*J. schimperiana*	200	15.3 ± 0.58^ac^	6.0 ± 0.0
*J. schimperiana*	100	13.3 ± 1.15^ac^	6.0 ± 0.0
*J. schimperiana*	50	10.67 ± 0.58^a^	6.0 ± 0.0
Ketoconazole	15 *µ*g	30.75 ± 1.70	23.5 ± 1.29
DMSO	5%	6.0 ± 0.0	—
Tween 80	5%	—	6.0 ± 0.0

Values are expressed as the mean ± SD. ^a^Significance difference (*p* < 0.05) compared to negative control; ^b^significance difference (*p* < 0.05) of 200 mg/mL compared to 50 mg/mL and 100 mg/mL; ^c^significance difference (*p* < 0.05) of 200 mg/mL and 100 mg/mL compared to 50 mg/mL. 6.0 ± 0.0 indicates that there is no inhibition zone created (it is considered as negative).

## Data Availability

The first author and corresponding author can supply all data used in the study.
